# Viscoelastic Properties of ECM-Rich Embryonic Microenvironments

**DOI:** 10.3389/fcell.2020.00674

**Published:** 2020-08-31

**Authors:** Zsuzsa Akos, Dona Greta Isai, Sheeja Rajasingh, Edina Kosa, Saba Ghazvini, Prajnaparamita Dhar, Andras Czirok

**Affiliations:** ^1^Division of Biology and Biological Engineering, California Institute of Technology, Pasadena, CA, United States; ^2^Department of Anatomy & Cell Biology, University of Kansas Medical Center, Kansas City, KS, United States; ^3^Department of Bioscience Research, University of Tennessee Health Science Center, Memphis, TN, United States; ^4^Department of Research, Kansas City University of Medicine and Biosciences, Kansas City, MO, United States; ^5^Chemical & Petroleum Engineering, The University of Kansas, Lawrence, KS, United States; ^6^Department of Biological Physics, Eotvos University, Budapest, Hungary

**Keywords:** ECM, quail embryo, microrheology, elasticity, Young's modulus, Zener solid, magnetic, nanorods

## Abstract

The material properties of tissues and their mechanical state is an important factor in development, disease, regenerative medicine and tissue engineering. Here we describe a microrheological measurement technique utilizing aggregates of microinjected ferromagnetic nickel particles to probe the viscoelastic properties of embryonic tissues. Quail embryos were cultured in a plastic incubator chamber located at the center of two pairs of crossed electromagnets. We found a pronounced viscoelastic behavior within the ECM-rich region separating the mesoderm and endoderm in Hamburger Hamilton stage 10 quail embryos, consistent with a Zener (standard generalized solid) model. The viscoelastic response is about 45% of the total response, with a characteristic relaxation time of 1.3 s.

## 1. Introduction

Tissues are physical bodies, thus their formation necessarily involves controlled generation and relaxation of mechanical stresses (Preziosi et al., 2010). Tissue cells are known to generate mechanical stresses by actin-myosin contractility, specifically relying on non-muscle Myosin II, with upstream regulators coordinated through a spatial and temporal activity of rho GTPases such as RhoA (Ridley et al., 2003). The relaxation of mechanical stresses involves the disruption of cell-cell connections, often accompanied by changes in cell neighbors (Forgacs et al., 1998; Smutny et al., 2017; Petridou et al., 2019). While this process is less understood on the molecular level than acto-myosin contractility, the spatio-temporal regulation for both force generation and relaxation are equally important to shape the embryonic tissues. Embryonic tissues are thus plastic, with their stress-free shapes deforming through the development process.

A cell-resolved mechanism underlying tissue plasticity was first resolved in flies, where studies indicated a pulsatile, ratchet-like contraction mechanism (Martin et al., 2009). Thus, instead of a uniformly distributed contractile activity across the tissue, individual cells were observed to undergo (asynchronously) a repeating cycle of contraction, stiffening and relaxation by cytoskeletal rearrangements. The pulsatile nature of tissue movements is also evident in the ECM displacements recorded within avian embryos (Szabó et al., 2011).

While measures for tissue deformation (strain) became recently possible to obtain during development (Rozbicki et al., 2015), estimates for tissue stress and material properties are still very challenging to determine. A FRET-based molecular sensor has been recently developed (Meng and Sachs, 2011) and used to measure tension *in vivo* (Cai et al., 2014), however its applicability in living tissues is still controversial (Eder et al., 2017). Instead, estimates of mechanical stress within tissues rely on mechanical perturbations (Hutson et al., 2003; Varner et al., 2010; Varner and Taber, 2012; Aleksandrova et al., 2015). In such experiments an introduced discontinuity alters the local mechanical balance of the tissue. As the tissue deforms to obtain a new mechanical equilibrium, this response can be recorded and evaluated. While precise stress measurements would require detailed knowledge about the spatial distribution of material parameters, such data are usually not available. Instead, the existence of tension or compression is deduced from the equilibrium shape of the wound (Varner et al., 2010); the wound opens up more if the stress component perpendicular to the cut is tensile.

The biophysical tool set measuring embryonic tissue rheology, however, is growing together with the interest to determine the material properties of the tissue (Petridou and Heisenberg, 2019). Microrheology, an especially promising approach, involves the analysis of the motion of colloidal tracer particles that are embedded into the sample of interest. The motion can be either a Brownian motion as in passive microrheology (Mason et al., 1997; Crocker et al., 2000; Baker et al., 2009), or driven by external forces as in active microrheology (Mizuno et al., 2008; Waigh, 2016; Vaclaw et al., 2018). These approaches can yield information on the local micro-mechanical properties (both viscous and elastic) of complex biopolymer networks like actin filaments, microtubules or intermediate filaments—both *in vitro*, and in live cells (Chen et al., 2010; Celedon et al., 2011; Nishizawa et al., 2017). The application of microrheology to extracellular matrix (ECM) materials has been rather limited so far (Waigh, 2016) and to the best of our knowledge has not been used to study the mechanical properties of cell-ECM assemblies that are of our interest. Yet, the ability to deduce the material properties prevalent in a microenvironment comparable with the size of the utilized probe, presents microrheology as a logical tool to explore tissues within a developing organism such as described in this study.

## 2. Methods

### 2.1. Nanorod Preparation

Nanorods 3 μm long and 300 nm in diameter were synthesized by electrochemical deposition of nickel into alumina templates as described previously (Paxton et al., 2004; Dhar et al., 2010; Ghazvini et al., 2015). The magnetized nickel nanorods were dispersed in a 90% isopropyl alcohol, 10% water solution.

### 2.2. Microrheology

**Figure 1:**
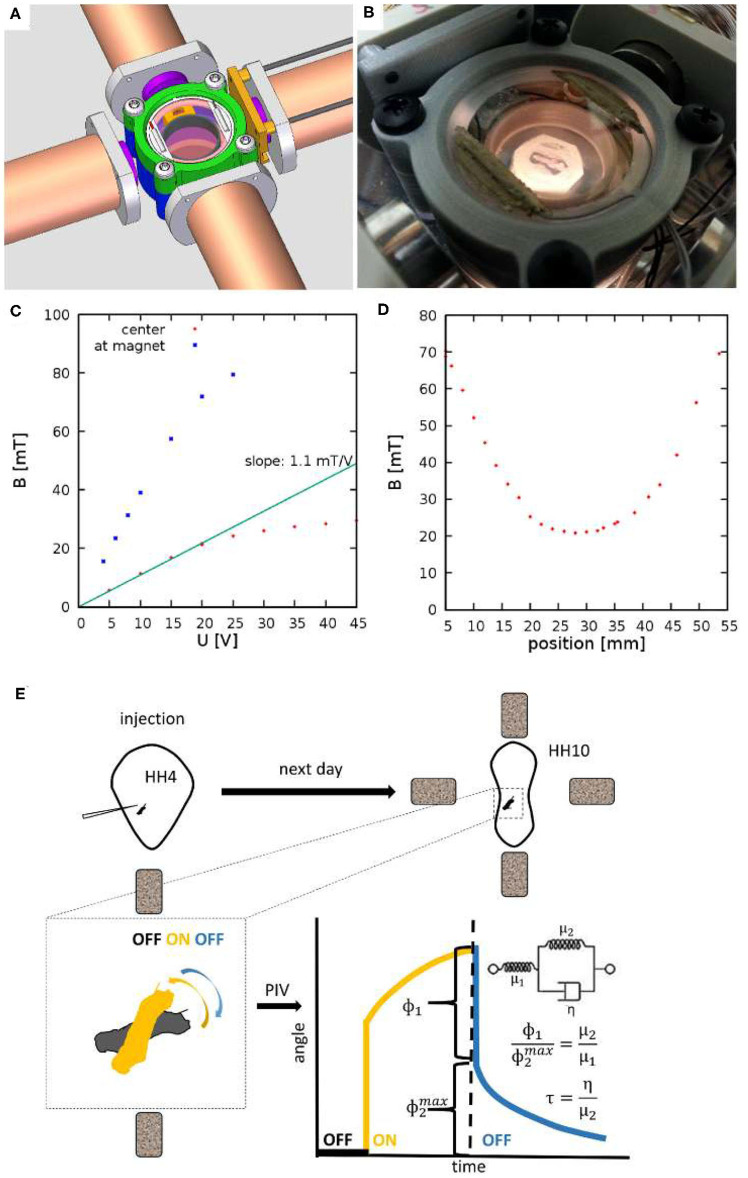
Experimental setup to measure viscoelastic properties of embryonic tissues. **(A)** CAD drawing showing four electromagnets and a 3D-printed incubator chamber in the center. **(B)** The incubator is heated by ITO coated glass windows at the top and bottom of the chamber. **(C)** The measured magnetic field as a function of the voltage across the electromagnets. Red and Blue symbols indicate measured values at the center of the incubation chamber and in the proximity of an iron core, respectively. **(D)** Spatial profile of the magnetic field, measured at *U* = 20V. **(E)** Schematic of measurement: HH Stage 4 quail embryos are microinjected with ferromagnetic nanorod aggregates. After an overnight incubation the specimen is placed between the electromagnets. Switching the magnetic fields on and off exerts a torque on the aggregate. Its rotation and that of the adjacent tissue is recorded by live imaging. Angle of rotation, as a function of time, was extracted using a Particle Image Velocimetry (PIV) method. The rotation response is evaluated in terms of a standard linear solid model, as described in the text.

For magnetic microrheology we have custom built electromagnets (Figure 1A) using 5 inches long iron cores (Ed Fagan Inc., alloy 79, 0.750^′′^ diameter) wrapped around with multiple layers of magnet wire (Tech Fixx Inc., 22 awg).

The theory of elasticity measurement follows (Wilhelm et al., 2002; Celedon et al., 2011). Let ϕ and θ denote the direction of the magnetic moment of the particle and the external field in the xy plane, respectively. The torque *T*_*magnetic*_ of the magnetic field *B*_0_ acting on a particle with magnetization *m* is

(1) $$\begin{array}{r} T_{magnetic}=mB_{0}\sin\left(\theta -\phi \right). \end{array}$$

Within an elastic material, the torque *T*_*elastic*_ resisting the rotation of the particle in the x-y plane is

(2) $$\begin{array}{r} T_{elastic}=-\mu f\left(\phi -\phi_{0}\right) \end{array}$$

where μ = *E*/[2(1 + ν)] is the shear modulus, *f* = πℓ^3^/[3ln(ℓ/4*r*)] is a geometric factor and ϕ_0_ denotes the particle's direction in the absence of external forces or fields (Wilhelm et al., 2002; Celedon et al., 2011). Similarly, the torque associated with a viscous drag is

(3) $$\begin{array}{r} T_{visc}=-\eta f\dot{\phi } \end{array}$$

where η is the viscosity, and $\dot{\phi }$ is the angular velocity of the nanorod.

In the Kelvin-representation of the standard linear solid (SLS), an elastic and a Kelvin-Voigt material are in series: the short term response is hence elastic, followed by a slower viscoelastic relaxation to a new elastic equilibrium. In this approximation the rotation of the material Δϕ = ϕ−ϕ_0_ is decomposed into the sum Δϕ = ϕ_1_ + ϕ_2_, where the terms indicate the initial elastic and the subsequent viscoelastic responses, respectively. Thus, the torque balance for a magnetic particle embedded in an SLS material is

(4) $$\begin{array}{r} T_{magnetic}=\mu_{1}f\phi_{1}=\mu_{2}f\phi_{2}+\eta f\dot{\phi_{2}} \end{array}$$

For small deformations Δϕ ≪ 1, we approximate *T*_*magnetic*_ as a Taylor series:

(5) $$\begin{array}{r} T_{magnetic}=mB_{0}\sin\left(\theta -\phi_{0}\right)-mB_{0}\cos\left(\theta -\phi_{0}\right)\Delta \phi +... \end{array}$$

Unless ϕ_0_ and θ are parallel, |sin(θ−ϕ_0_)|≫|cos(θ−ϕ_0_)Δϕ|, thus *T*_*magnetic*_ remains a constant during small deformations. Under such conditions, the solutions of Equation (4) are:

(6) $$\begin{array}{r} \phi_{1}=\frac{T_{magnetic}}{\mu_{1}f} \end{array}$$

and

(7) $$\begin{array}{r} \phi_{2}=A\left[1-\exp\left(-t/\tau \right)\right] \end{array}$$

where *A* = *T*_*magnetic*_/(μ_2_*f*) and the characteristic relaxation time is

(8) $$\begin{array}{r} \tau =\eta /\mu_{2}. \end{array}$$

In the steady state elastic torques resists all the externally imposed *T*_*magnetic*_ in the Kelvin-Voigt unit, hence

(9) $$\begin{array}{r} T_{magnetic}=\mu_{2}f\phi_{2}^{max}. \end{array}$$

Thus, the ratio of the viscoelastic and pure elastic response is obtained as

(10) $$\begin{array}{r} \frac{\phi_{1}}{\phi_{2}^{max}}=\frac{\mu_{2}}{\mu_{1}}. \end{array}$$

### 2.3. Embryo Culture

Fertile wild type quail (Coturnix coturnix japonica) eggs (Ozark Egg Co., Stover, MO) were incubated for varying periods of time (from 20 to 36 h) at 37°C to reach Hamburger and Hamilton (HH) stage 4 (Hamburger and Hamilton, 1951). Embryos were then isolated, injected and cultured as in (Aleksandrova et al., 2015), modified from (Chapman et al., 2001) to reach HH10 when they were subjected to experimental analysis.

### 2.4. Microinjection and ECM Labeling

Monoclonal antibodies directed against fibrillin-2 and fibronectin ECM proteins (JB3, B3D6; DSHB, Iowa City, IA) were directly conjugated to AlexaFluor 488, 555, or 647 (Molecular Probes) according to the manufacturer's instructions (Czirok et al., 2006). The direct conjugates were injected into the lateral plate mesoderm as 5–40 nl boluses using a PLI-100 (Harvard Instruments) microinjector as described in Little and Drake (2000). Microinjections were performed 30–60 min prior to the beginning of the image acquisition to allow for antibody diffusion and antigen binding.

### 2.5. Preparation of Transverse Plastic Sections

The embryos were dehydrated through graded ethanol series, placed in acrylamide containing infiltration solution for an hour under vacuum, and embedded in an acrylamide-agarose resin. Subsequently, 100 um sections were cut using a vibratome (Germroth et al., 1995).

### 2.6. Microscopy

Microrheological measurements were performed on the powered stage of a dissecting microscope (Leica M205FA) equipped with epifluorescence illumination and a Planapo 2.0x objective. The imaging system recorded 1,392 × 1,040 pixel images at a rate of 15.44 frames/s and at a resolution of 0.4 μm /pixel.

### 2.7. Optical Flow-Based Analysis of Local Tissue Rotation

To characterize tissue deformation, we first apply our non-invasive, optical flow-based method described in Czirok et al. (2017) for each image of the recording. The displacement field $\vec{u}\left(t,\vec{x}\right)$, calculated relative to the first image as a reference, provides the basis to calculate local tissue rotation. We approximate the local vorticity as

(11) $$\begin{array}{r} |\nabla \times \vec{u}\left(t,\vec{x}\right)|=\frac{\partial u_{y}}{\partial x}-\frac{\partial u_{x}}{\partial y}\\ \;\approx \frac{u_{y}\left(x+h,y\right)-u_{y}\left(x-h,y\right)-u_{x}\left(x,y+h\right)+u_{x}\left(x,y-h\right)}{2h} \end{array}$$

where *h* is the resolution of the optical flow-derived grid.

## 3. Results

### 3.1. Magnetic Microrheometer

To facilitate microrheology measurements in live embryos, we built a plastic incubator chamber surrounded by two, orthogonal pairs of electromagnets (Figure 1). The plastic construction of the incubator chamber minimizes perturbations of the magnetic field. The incubator chamber consists of two heated indium tin oxide (ITO) glass surfaces that enclose a 35mm dish (Figures 1A,B). In the dish a 3D-printed ring (Gulyas et al., 2018) delineates an inner chamber, filled by low melting point agarose, while the outer chamber is filled with sterile distilled water to provide humidity. Temperature was controlled by heating currents within the ITO surfaces, feedback was provided by a thermometer probe immersed in the water bath surrounding the agarose bed. Quail embryos were cultured at the surface of the agarose bed.

The magnetic field within the incubator chamber could be gradually adjusted up to a value of 30 mT by setting the voltage across the electromagnets (Figure 1C). The approximate Helmholtz pair-like configuration of the electromagnets was designed to provide a spatial homogeneous magnetic field. According to our measurements, within a 10 mm diameter region around the symmetry center the magnetic field changes less than 5% (Figure 1D).

### 3.2. Microinjection of Ferromagnetic Nickel Nanorod Probes

**Figure 2:**
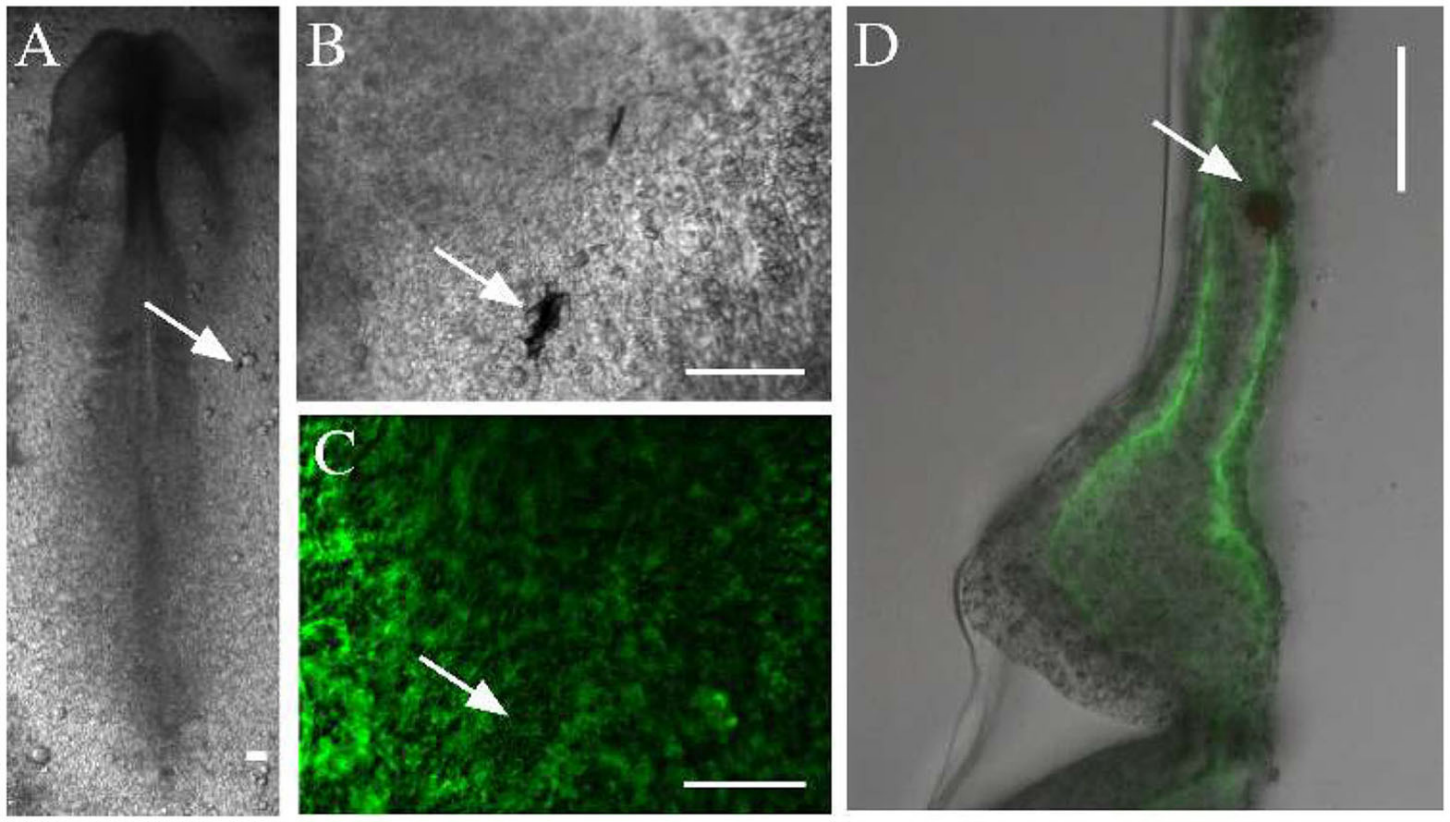
Microinjected ferromagnetic rod aggregates in quail embryos. **(A,B)** An embryo, microinjected with ferromagnetic aggregates at HH stage 4 and healed during an overnight incubation (shown at HH stage 7). Scale bars indicate 100 μm, white arrows point to the same aggregate. **(C)** The ECM microenvironment is visualized by fluorescently labeled antibodies (JB3 anti-fibrillin, B3D6 anti-fibronectin mixture) microinjected into the extracellular space. **(D)** A 100 μm thick transverse cross section of the same embryo locates the aggregate between the endoderm and the lateral plate mesoderm.

To measure the material properties of embryonic tissues, we microinjected ferromagnetic nanorods into HH stage 4 quail embryos. In the confined space of the injector capillary, the particles formed aggregates, which incorporated into the tissue, and were detectable by transmitted light microscopy for the entire length of ex ovo development (Figures 2A,B). The aggregates also appear as dark areas against the background of ECM immunofluorescence (Figure 2C). As subsequent physical sectioning of the microinjected embryos revealed, most nanorod aggregates were delivered into the ECM rich space separating the mesoderm and the endoderm (Figure 2D).

### 3.3. Tissue Deformation Forced by External Fields

**Figure 3:**
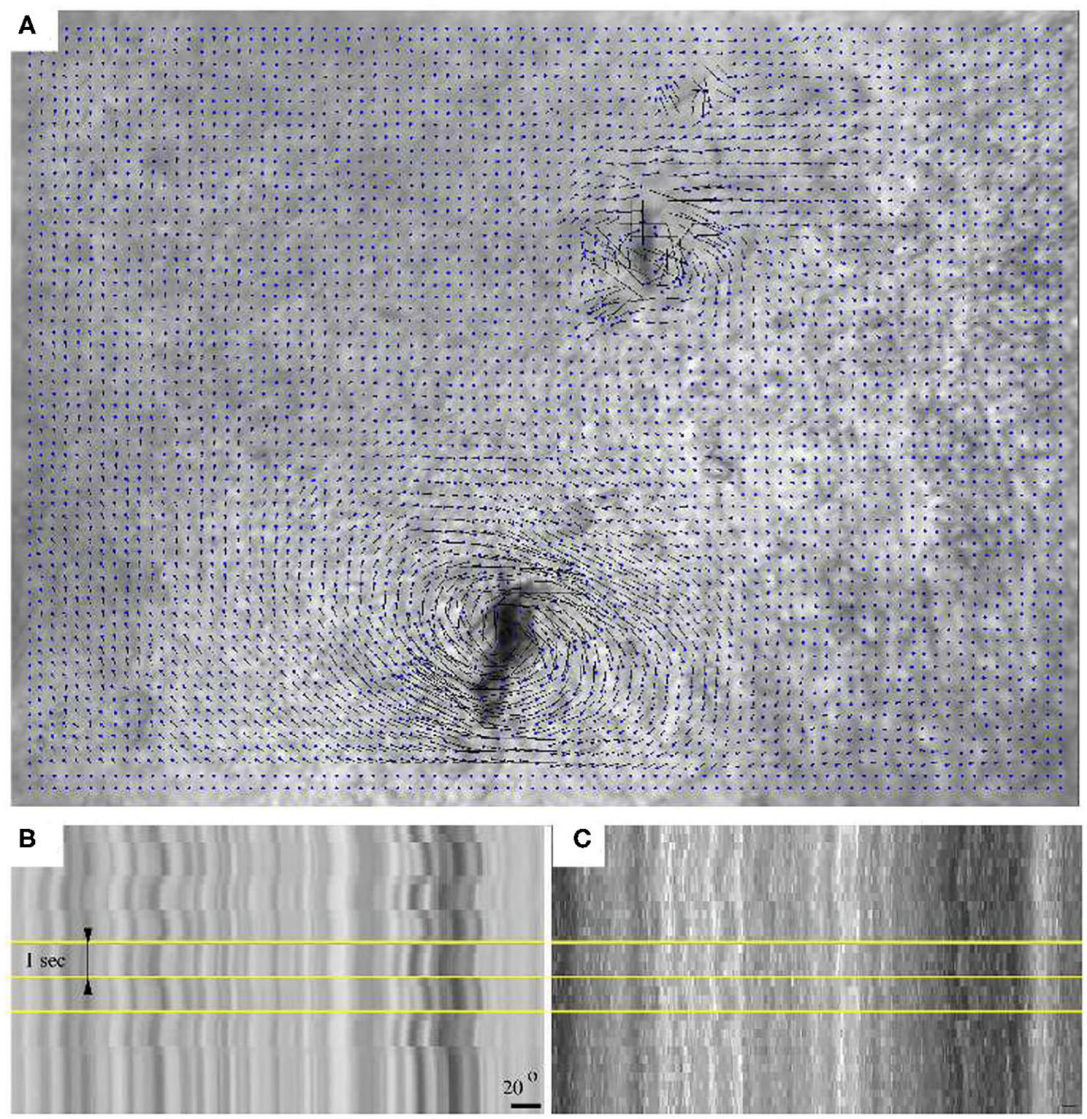
Switching the direction of the external magnetic field rotates an aggregate of magnetic rods and the tissue environment within a HH 10 quail embryo. **(A)** Tissue displacement, calculated using Particle Image Velocimetry (PIV). **(B)** A kymograph representation of the tissue movements reveals the extent of magnetic field-induced rotation. **(C)** Kymograph of the corresponding immunofluorescence recording. Horizontal yellow lines indicate changes in magnetic field direction, timed at 1 s intervals. The scale bar indicates a rotation of 20°.

Microrheological recordings were performed in HH10 embryos—by which time the injury associated with microinjection completely healed. As high framerate transmitted light live imaging reveals, alternating magnetic fields readily induce rotation of the embedded aggregates, accompanied by a profound deformation of the surrounding tissue microenvironment (Figure 3, Supplementary Movie 1). The deformation of the ECM was established by live imaging of fibronectin and fibrillin immunofluorescence (Supplementary Movie 2, Figure 2C). As kymogrphs demonstrate by visualizing movement along the perimeter of a 50 μm radius circle centered at an aggregate, the external force-induced deformation of the ECM and the tissue was similar both in magnitude and timing (Figures 3B,C).

**Figure 4:**
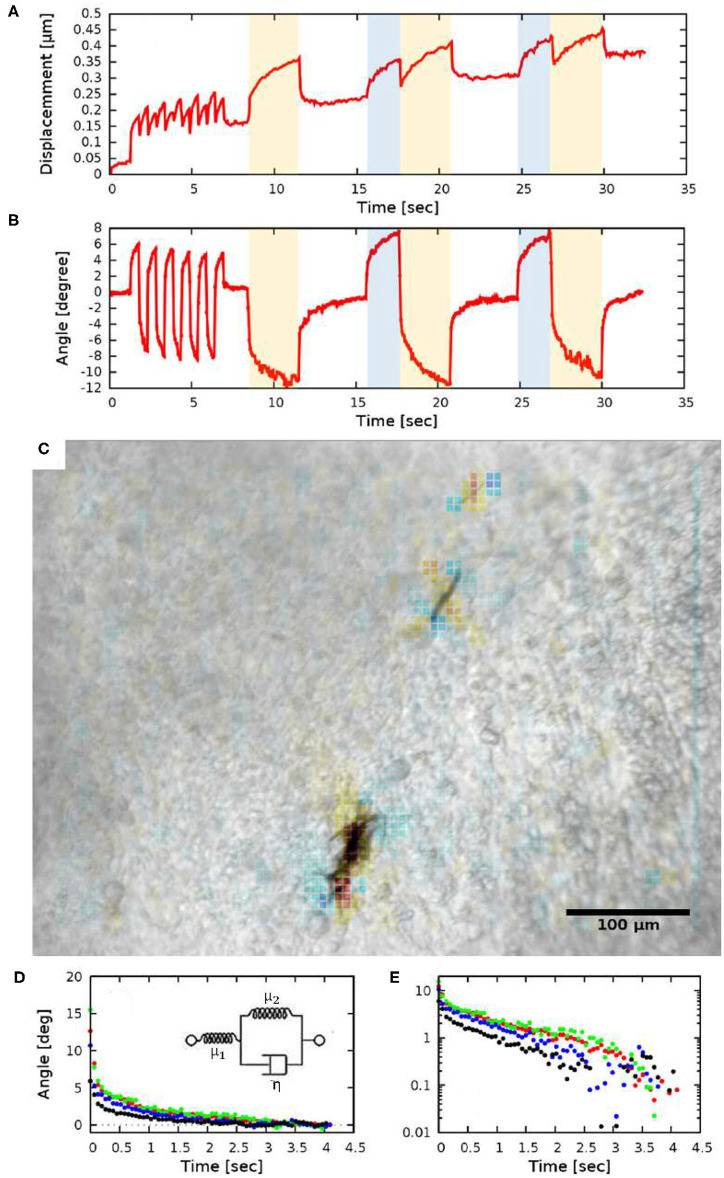
Quantitative measures of tissue rotation obtained from live recordings. **(A)** Average displacements relative to a reference frame. Shaded areas indicate the time while the electromagnets were turned on (the two orthogonal electromagnet pairs are indicated with distinct colors, blue and yellow). **(B)** Angle of rotation calculated from the vorticity (curl) of the displacement field. **(C)** Vorticity of the PIV displacement field, superimposed on a corresponding brightfield image. **(D,E)** Viscoelastic creep of the embryonic tissue. Difference between the current angle and the estimated equilibrium value |ϕ(*t*)−ϕ_∞_|, as a function of time elapsed since the switch in magnetic field direction. The distinct colors indicate four HH 10 embryos, each injected at the lateral plate mesoderm. The data set presented with black symbols was obtained with a magnetic field of 21 mT (2.35 A coil current), while the other data sets were obtained using a magnetic field of 26 mT (3.4 A coil current). The exponential decay on a linear axis **(D)** appear as straight lines on a logarithmic axis **(E)**. The observed behavior is consistent with a Zener solid (inset **D**).

To quantify the tissue deformations induced by the rotation of ferromagnetic aggregates, we modified our image analysis tools used to characterize cardiomyocyte beating activity (Czirok et al., 2017). We compared a sequence of images to a common reference frame by PIV analysis, yielding a displacement field (*u*). The time-dependent spatial average of *u* indicates a gradually increasing baseline, upon which the magnetic field-induced changes are superimposed (Figure 4A). The increasing baseline reflects deformations intrinsic to the developing tissue.

Tissue rotation was specifically characterized by calculating vorticity (Figure 4C), the amount of local spinning motion that would be seen by a local observer moving with the tissue. The overall rotation was established based on Stokes' theorem: the sum total of vorticity within an area gives the amount of circulation along the perimeter. Thus, by calculating the sum of vorticity over circles of various sizes, we can determine the spatial extent of the tissue deformation as well as the magnitude of the rotation (Figure 4B).

While we do not know the net magnetic moment of the aggregates, the temporal behavior of tissue rotation allows the characterization of the local viscoelastic response of the tissue using Equations (7) and (8). As Figure 4B shows, the response of the tissue is biphasic: a very fast (less than 0.2 s) adjustment is followed by a slow, creep-like behavior lasting for several seconds. As a quantitative measure of the response, we fitted an exponential function

(12) $$\begin{array}{r} \phi \left(t\right)=a\exp\left(-t/\tau \right)+\phi_{\infty } \end{array}$$

to each of the recorded responses—both in the creep and relaxation phases—and then transformed the data so that the asymptotic value ϕ_∞_ was shifted to zero. The average time-dependent difference from the estimated equilibrium value |ϕ(*t*) − ϕ_∞_| indeed validates the presence of a slow, exponential relaxation with a characteristic time of 1.3 ± 0.2 s (Figures 4D,E). The presence of a faster and a slower response thus suggest that the ECM-containing early embryonic tissue is well-described as a Zener material (Mainardi and Spada, 2011), represented with a spring in series with a Kelvin-Voigt unit (Figure 4D inset). By fitting the Zener model to data extracted from HH10 embryos (*n* = 4) we calculated μ_2_/μ_1_ = 0.45 ± 0.1 and found that the ratio of the viscoelastic and pure elastic response is 45:55%.

## 4. Discussion

Compression of cell aggregates yielded the first insight into the viscoelastic properties of cell assemblies (Forgacs et al., 1998; Khalilgharibi et al., 2016). These studies established a biphasic elastoplastic response: when aggregates are compressed, there is an initial reversible elastic deformation. When the compressed state is sustained, the forces required to maintain the deformation diminish. For most cell types the force relaxation exhibits an initial fast decay with a characteristic time of around 2 s. This initial decay is followed by a slower exponential process with a characteristic time of 20 s. This late stage process involves a plastic change of the stress free shape of the aggregate: when the external compression is removed, the aggregates did not return to their initial spherical shape for almost a day. The plastic deformation is accompanied by cellular rearrangement in the bulk: by exchanging neighbors cells restored their cuboidal shape. Our measurements remained in the elastic regime: the stress free state of the tissue did not change as evidenced by the diminishing rotation angle upon turning the external magnetic fields off. The tissue response, however, was viscoelastic: an initial elastic response followed by an exponential creep. The characteristic time scale of the creep was consistent with the time scale of the fast phase in (Forgacs et al., 1998). We suspect that the viscous component arises by movements of cytoskeletal and ECM components in the presence of drag forces from the cytosol and the interstitial fluid inside and outside of the cells, respectively.

Our measurements did not cover the plastic regime as at longer time scales tissue deformations intrinsic to developmental processes interfere with the analysis. The microaspiration technique on *Xenopus laevis* embryos measure material properties on larger scales, and found power law stress relaxation (von Dassow et al., 2010), i.e., a remodeling process fundamentally slower than those found in cell aggregates. Interestingly the creep response was still linear: thus no evidence for active mechanical feedback was observed.

Previous measurement on the chick embryo lateral plate mesoderm found *E* = 1, 300 Pa for the Young's modulus when evaluated the tissue deformation caused by a cantilever beam (Agero et al., 2010). This value, together with a Poisson number of 0.2 (Wilhelm et al., 2002; Celedon et al., 2011) yields a shear modulus μ_1_ + μ_2_ = *E*/2.4 ≈ 550 Pa. Thus, from our measurements μ_2_ ≈ 250Pa and η = μ_2_τ ≈ 300 Pa s, a value consistent with behavior observed in ECM hydrogels *in vitro* (Massensini et al., 2015).

Since cantilever beam probing encompasses a larger area, a more local measurement would be useful to determine local material parameters (*E* and η) inside the embryo. This could be potentially achieved with the same method presented here, but using magnetic beads where magnetization can be determined and small enough to inject. Further studies can take this direction to explore additional local internal tissue properties.

The importance of tissue material properties on stem cell differentiation (Charrier et al., 2018) generated renewed interest in the mechanical testing of the embryonic (D'Angelo et al., 2019) and organotypic tissues (Chevalier et al., 2016; Charrier et al., 2018) and cells. We trust that the magnetic microrheology method reported here will be a valuable tool to probe tissues at the intermediate length scales, between that of cells and whole organs.

## Data Availability Statement

The datasets generated for this study are available on request to the corresponding author.

## Author Contributions

ZA, SG, PD, and AC designed experiments and the microrheological instrument. PD provided nanorods and designed the calibration experiment. ZA, SG, and EK performed the calibration. EK and SR injected quail embryos and performed measurements. ZA, DI, and AC analyzed data and wrote the manuscript.

## Conflict of Interest

The authors declare that the research was conducted in the absence of any commercial or financial relationships that could be construed as a potential conflict of interest.
